# Prolate spheroidal hematite particles equatorially belt with drug-carrying layered double hydroxide disks: Ring Nebula-like nanocomposites

**DOI:** 10.1186/1556-276X-6-116

**Published:** 2011-02-03

**Authors:** Ahmet Nedim Ay, Deniz Konuk, Birgul Zümreoglu-Karan

**Affiliations:** 1 Department of Chemistry, Hacettepe University, Beytepe Campus, 06800 Ankara, Turkey

## Abstract

A new nanocomposite architecture is reported which combines prolate spheroidal hematite nanoparticles with drug-carrying layered double hydroxide [LDH] disks in a single structure. Spindle-shaped hematite nanoparticles with average length of 225 nm and width of 75 nm were obtained by thermal decomposition of hydrothermally synthesized hematite. The particles were first coated with Mg-Al-NO_3_-LDH shell and then subjected to anion exchange with salicylate ions. The resulting bio-nanohybrid displayed a close structural resemblance to that of the Ring Nebula. Scanning electron microscope and transmission electron microscopy images showed that the LDH disks are stacked around the equatorial part of the ellipsoid extending along the main axis. This geometry possesses great structural tunability as the composition of the LDH and the nature of the interlayer region can be tailored and lead to novel applications in areas ranging from functional materials to medicine by encapsulating various guest molecules.

## Introduction

Magnetic iron oxide nanoparticles have attracted extensive attention in biomedicine and nanotechnology areas [1,2]. Among them, hematite (α-Fe_2_O_3_) is the oldest known, most stable, and cheapest iron oxide with n-type semiconducting and soft magnetic properties [3]. Since the report of Matijevic and co-workers in the early 1980s [4], much progress has been made toward the synthesis of monodisperse hematite particles with many different shapes that offer promising uses in water splitting, photocatalysis, photoelectrochemistry, magnetic recording media, and other nanodevices [5-7].

For practical applications, magnetic nanoparticles are coated with a protective shell to avoid agglomerization and for chemical stabilization [8]. A nonmagnetic coating is generally employed not only for magnetic core stabilization but also for the integration of biofunctionalization [9]. So far, many spherical core-shell magnetic nanostructures have been reported, while non-spherical core-shell particles with lower symmetries are relatively rare, although they would offer interesting physical properties. Ellipsoidal particles may serve as simple non-spherical models for studying anisotropic optoelectronic effects and drug delivery [10,11]. There has been considerable interest in the synthesis and characterization of non-spherical hybrid nanostructures prepared by coating spindle-shaped hematite particles with gold [12], silica [13], titania [14], and polymeric shells [15].

LDHs have been introduced as alternative inorganic coating materials for magnetic nanoparticles [16]. A number of magnetic core@LDH nanohybrids have been synthesized for catalysis [17,18] and drug delivery [19-21] applications. We have recently reported anti-arthritic agent-carrying, nearly spherical core-shell magnesium ferrite@LDH nanocomposites that have a potential for magnetic arthritis therapy [22]. In this communication, we describe an original morphology of such nanocomposites using spindle-shaped hematite as the core material and salicylate-intercalated Mg-Al-LDH as the shell.

## Experimental details

Hematite nanoparticles were obtained by thermal decomposition of iron(III) oxalate in static air. Iron(III) oxalate was prepared hydrothermally by treating aqueous FeCl_3 _and H_2_C_2_O_4 _at pH 7 (adjusted by ammonia solution) for 48 h at 80°C in a pressure bomb in the presence of a cationic surfactant (cetyl tributyl ammonium bromide). The product was washed thoroughly several times with water and dried at room temperature. The powder was ground in an agate mortar and calcined at 300°C for 6 h.

Element analysis for metal ions was performed using a Spectro XLAP 2000 PRO XRF X-ray fluorescence spectrometer (Spectro Analytical Instruments GmbH) while for carbon and hydrogen on a varioMICRO CHNS instrument (Elementar Analysensysteme GmbH). The water content was determined by thermogravimetry on a DTG-60H (Shimadzu) thermal analysis system at a heating rate of 10°C/min. Powder X-ray diffraction patterns [XRD] were recorded using a D/MAX-2200 (Rigaku) diffractometer equipped with graphite-filtered Cu Kα radiation (*λ *= 1.54056 Å) from 3° to 70° (2*θ*) at a scanning rate of 4 min^-1^. Fourier transform infrared spectra [FTIR] were recorded in the range from 4,000 to 400 cm^-1 ^on a Perkin Elmer Spectrum One instrument using the KBr pellet technique. The morphology and dimension of the synthesized products were observed with a FEI quanta 200 FEG (FEI Company) scanning electron microscope [SEM]. Transmission electron microscopy [TEM] and selected area electron diffraction [SAED] were performed using a FEI Tecnai G2 F30 (FEI Company) instrument operated at 300 or 100 kV. Magnetism of the products was measured at room temperature with a vibrating sample magnetometer (Quantum Designed Physical Property Measurement System (Quantum Design Inc.) in the magnetic field range of ±30 kOe. The electronic spectra were recorded on a Shimadzu UV-3600/UV-VIS-NIR Spectrophotometer (Shimadzu) equipped with a Praying Mantis attachment.

## Results and discussion

**Figure 1:**
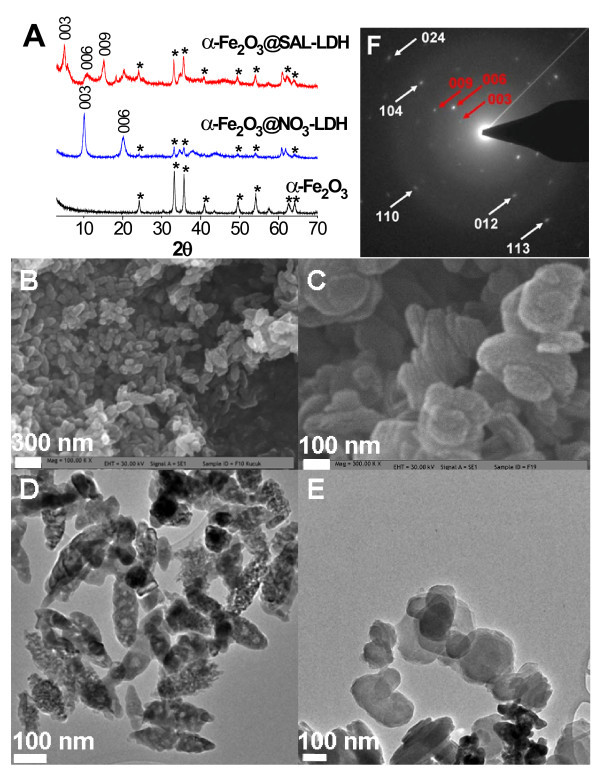
**Powder X-ray diffraction patterns, SEM and TEM images of the as-prepared samples**. XRD patterns of uncoated, NO_3_-LDH-coated, and SAL-LDH-coated hematite (**A**). SEM images of uncoated (**B**) and SAL-LDH-coated hematite (**C**). TEM images of uncoated (**D**) and SAL-LDH-coated hematite (**E**). SAED pattern of SAL-LDH-coated hematite (**F**).

Figure 1a shows the powder X-ray diffraction pattern of the as-prepared hematite sample. The pattern indicates single phase of α-Fe_2_O_3 _with characteristic sharp reflections at *d *values of 3.66 Å (012), 2.69 Å (104), 2.51 Å (110), 2.20 Å (113), 1.83 Å (024), 1.69 Å (116), 1.48 Å (214), and 1.45 Å (300), matching with the JCPDS file 13-534. The FTIR spectrum confirmed the hematite structure with two characteristic bands located at 547 and 478 cm^-1 ^[23]. TEM and SEM images of the as-synthesized hematite nanoparticles showed a well-defined spindle morphology with a mean edge length in the range from 200 to 220 nm and edge width from 70 to 80 nm; the length-to-width ratio is about 3 (Figure 1B-E).

Hematite particles were then coated with Mg-Al-NO_3_-LDH, as described previously for MgFe_2_O_4_@NO_3_-LDH [22]. The XRD pattern of the as-prepared α-Fe_2_O_3_@NO_3_-LDH nanohybrid displayed typical *d*_003 _and *d*_006 _reflections due to the presence of the LDH shell, while characteristic peaks of the core materials (indicated by an asterisk in Figure 1A) remained intact during the coating process. From the spacing for the *d*_003 _reflection, the interlayer distance was calculated as 8.7 Å. α-Fe_2_O_3_@NO_3_-LDH particles were then treated with acetylsalicylic acid solution, which gave salicylate ions (SAL, C_7_H_5_O_3_) by hydrolysis at alkaline reaction conditions. As *in vivo *salicylate is approximately equipotent to aspirin [24], the exchange of interlayer nitrate ions with salicylate ions resulted in the formation of a new bio-nanohybrid: α-Fe_2_O_3_@SAL-LDH. Intercalation of salicylate into the LDH structure was clearly followed as the *d*_003 _and *d*_006 _reflections for the NO_3_-LDH disappeared; thereby, a new series of intense basal reflections at lower 2*θ *values appeared instead. The basal spacing of the LDH increased from 8.7 to 17.2 Å owing to the incorporation of the larger organic ion between the layers. Figure 1F shows the SAED pattern of the final nanocomposite. The pattern was solved and diffraction spots from the LDH phase were indicated by red arrows while those from the core phase indicated by white arrows, confirming that the core particles were covered by the LDH shell.

**Figure 2:**
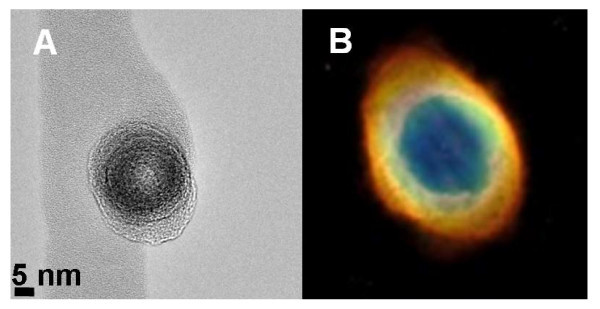
**Morphological resemblance of the as-prepared nanocomposite to The Ring Nebula**. TEM image of α-Fe_2_O_3_@SAL-LDH (end-on view) (**A**). The Ring Nebula (end-on view; Credit: Hubble Heritage, http://www.nasa.gov) (**B**)

The anisotropic morphology of the α-Fe_2_O_3_@SAL-LDH particles was revealed by SEM and TEM analyses. It is clearly seen from the SEM image that LDH disks are stacked parallel to the short axis and extend along the long axis of the prolate spheroidal α-Fe_2_O_3 _core, giving rise to a heterostructured nanohybrid (Figure 1C). This structural feature has a unique resemblance to that of the Ring Nebula, which is 2,000 light years away from our planet. This Nebula has thick equatorial rings extending through its main axis of symmetry. It appears to be a non-spherical planetary nebula with strong concentrations material around the waist (Figure 2).

**Figure 3:**
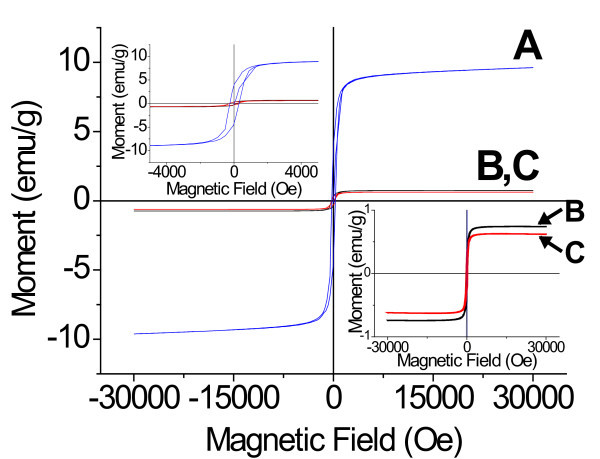
**Room temperature magnetization curves of uncoated and coated hematite particles**. Uncoated (**A**), NO_3_-LDH-coated (**B**), and SAL-LDH-coated hematite (**C**).

Figure 3 shows the room temperature magnetization curves of uncoated and coated hematite particles. The observed narrow hysteresis loops (shown in the inset) with small coercivity and remanence magnetization behavior are characteristics of a soft ferromagnet [25]. The measured saturation magnetization values for α-Fe_2_O_3_@NO_3_-LDH (0.7 emu/g) and α-Fe_2_O_3_@SAL-LDH (0.6 emu/g) were lower than that of the naked hematite (9.6 emu/g). The decreased saturation magnetization should be attributed to the presence of the nonmagnetic material around the magnetic core and is related to the amount of the shell. α-Fe_2_O_3_@SAL-LDH was formulated as Fe_2_O_3_@4{Mg_0.68_Al_0.32_(OH)_2_(C_7_H_5_O_3_)_0.31_(NO_3_)_0.01_0.6H_2_O} using the chemical and thermogravimetric analysis data. The core content of the nanocomposite is 26 wt.% and the drug content 28 wt.%.

**Figure 4:**
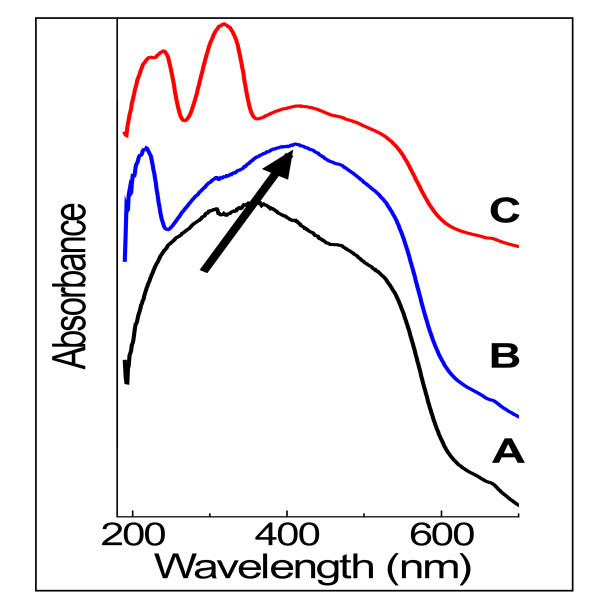
**Effect of LDH coating on the optical properties of hematite core**. Diffuse reflectance UV/Vis spectra of uncoated hematite (**A**), NO_3_-LDH-coated hematite (LDH peak at 218 nm) (**B**), and SAL-LDH-coated hematite (LDH and SAL peaks at 224, 240, and 319 nm) (**C**).

The effect of LDH coating on the optical properties of the hematite core is illustrated in Figure 4. Related to the change in morphology, ligand-to-metal charge transfer transition of the uncoated spindle hematite at 358 nm showed a red shift, while the shoulder due to the ligand field transition around 520 nm did not shift. This typical behavior for anisotropic hematite agrees with recent reports [26,27].

## Conclusion

In conclusion, we present here the first example of a non-spherical magnetic core@LDH shell architecture. This new structural feature is similar to that of the Ring Nebula, displaying a unique resemblance of nano to macro. The reported anisotropic nanohybrid possesses a great structural tunability and may show unprecedented properties in shape-sensitive drug delivery/release [28] and nanophotonics applications.

## Competing interests

The authors declare that they have no competing interests.

## Authors' contributions

ANA and DK carried out synthesis and characterization studies. ANA and BZK performed data analysis and discussion of the results. BZK conceived of the study and wrote the manuscript. All authors read and approved the final manuscript.
